# miR-200b as a prognostic factor in breast cancer targets multiple members of RAB family

**DOI:** 10.1186/1479-5876-12-17

**Published:** 2014-01-21

**Authors:** Feng Ye, Hailin Tang, Qing Liu, Xinhua Xie, Minqing Wu, Xiaoping Liu, Bo Chen, Xiaoming Xie

**Affiliations:** 1 Department of Breast Oncology, Sun Yat-Sen University Cancer Center, 651 East Dongfeng Road, Guangzhou, Guangdong 510060, People’s Republic of China; 2 State Key Laboratory of Oncology in South China, Sun Yat-Sen University Cancer Center, Collaborative innovation center for cancer medicine, 651 East Dongfeng Road, Guangzhou, Guangdong 510060, People’s Republic of China; 3 The Center for Skull Base Surgery and Neurooncology, Changsha, Hunan, People’s Republic of China

**Keywords:** miR-200b, RAB family, Breast cancer, Prognosis

## Abstract

**Background:**

miR-200b has been reported to be a tumor suppressor and a promising therapeutic target in cancer. miR-200b has been associated with epithelial-mesenchymal transition and chemo-resistance in cancer. The aim of this study is to investigate the expression of miR-200b, its prognostic roles and its potential targets in breast cancer.

**Methods:**

qRT-PCR was used to detect miR-200b expression in breast cancer tissues and cell lines. In situ hybridization of miR-200b on tissue microarray including 134 breast cancer samples was used to evaluate its prognostic role. Novel targets of miR-200b in breast cancer were predicted and confirmed by luciferase reporter assay and western bloting. Immunohistochemical staining was used for protein detection. The biological effects of miR-200b in breast cancer cells were further confirmed by ectopic expression of its mimics followed by MTT assay and invasion test.

**Results:**

miR-200b was downregulated in breast cancer tissues and cell lines and its low-expression correlated with poor outcome in breast cancer patients. Members of RAB family, RAB21, RAB23, RAB18 and RAB3B were predicted to be the targets of miR-200b. The luciferase reporter assay was performed to certificate this prediction. The expressions of RAB21, RAB23, RAB18 and RAB3B were suppressed by transfection of miR-200b in breast cancer cells. Over-expression of miR-200b or knock-down of RAB21, RAB23, RAB18 and RAB3B inhibited breast cancer cell proliferation and invasion in vitro.

**Conclusions:**

Our study provides evidence that miR-200b is a prognostic factor in breast cancer targeting multiple members of RAB family. MiR-200b could be a potential therapeutic target in breast cancer.

## Background

Breast cancer is the first ranked female malignancy worldwide, with about 200,000 new incidence and 40,000 deaths per year in US [1]. Generally breast cancers could be classified according to the TNM staging system [2] and their molecular groups [3], which include luminal A type (estrogen receptor (ER) + and/or progesterone receptor (PR) +, human epidermal growth factor receptor-2 (HER2)-), luminal B type (ER + and/or PR+, HER2+), HER2 over-expressing type (ER-, PR-, and HER2+), basal-like type (ER-, PR-, HER2-, cytokeratin 5/6+, and/or epidermal growth factor receptor (EGFR)+). Breast cancer of HER2 over-expressing or basal-like type would predict more recurrence, distant metastasis, and therapy resistance [4]. The comprehensive therapy and prognosis for breast cancer would depend on both the TNM stage and molecular subtype. Although breast cancers with early stage show excellent outcome after therapy, recurrent and metastatic breast cancer patients remain big problems for cure [5].

MicroRNAs, also termed miRNAs, are a class of endogenous, non-protein coding single-stranded RNA molecules with a length of 21–23 nucleotides, which plays a crucial role in the post-transcriptional regulation of gene expression [6]. miRNAs are highly conserved and specific, and regulate gene expression by binding to the 3′ untranslated region (UTR) of target messengerRNA (mRNAs) and inhibiting translation or inducing degradation of mRNAs. miRNAs has been proved to play vital roles in cancer management, acting as either oncogenes or tumor suppressors [7].

The miR-200 family (including miR-200a, miR-200b, miR-200c, miR-141 and miR-429) are transcribed from two chromosomal locations: miR-200b-200a-429 cluster at chromosomal location 1p36 and miR-200c-141 cluster at chromosomal location 12p13 [8]. Recently, the miR-200 family has been associated with carcinogenesis and cancer therapy [9]. Dysregulation of the miR-200 family was reported in several malignancies, including ovarian, endometrial, lung and gastric cancer [10-12]. The miR-200 family has been supposed to be tumor suppressors regulating epithelial-mesenchymal transition (EMT) [13]. miR-200 directly targets EMT-inducing transcriptional factors ZEB1 and ZEB2, which repress E-cadherin expression. miR-200 could also suppress β-catenin/Wnt signaling pathway by targeting β-catenin mRNA [14], thus highlighting its roles in cancer invasion and metastasis. Moreover, mi-200 has been reported to affect the chemotherapy and endocrine thrapy resistance in breast cancer [15,16]. Among the miR-200 family, miR-200b is thought to be the fundamental regulator in EMT and cancer chemo-sensitivity [17]. However, the expression of miR-200b and its prognostic role in breast cancer remain unclear.

In this study, we investigated the expression of miR-200b and its prognostic roles in breast cancer patients, predicted and further identified multiple members of RAB family as new targets of miR-200b in breast cancer.

## Materials and methods

### Cell lines and culture

Human mammary epithelial (HME) cell line 184A, MCF-10A, human breast cancer cell lines MDA-MB-231, MDA-MB-435, MCF-7, T47D, BT-474, BT-483, and mouse breast cancer cell line 4 T1 were obtained from the American Type Culture Collection (Manassas, VA, USA) and were passaged in our laboratory for less than six months after resuscitation of frozen aliquots. The breast cancer cells were cultured in Dulbecco’s modified Eagle’s medium (DMEM, Invitrogen, CA, USA) supplemented with 10% fetal bovine serum (FBS, GIBCO, Cappinas, Brazil), in a humidified incubator at 37°C containing 5% CO2. All cell lines were re-authenticated by short tandem repeat DNA profiling every 6 months after used.

### Quantitative real-time polymerase chain reaction analysis (qRT-PCR)

Expression level of miR-200b was detected in both breast cancer tissues and cell lines. 40 pairs of tumor tissues and para-carcinoma tissues from breast cancer patients were obtained. Human mammary epithelial cell line 184A, MCF-10A, and several breast cancer cell lines were also included. Total RNAs were extracted from tissues/cells with TRIzol reagent (Invitrogen). For miR-200b, reverse transcription and qRT-PCR reactions were performed by means of a SYBR-green-containing PCR kit (GenePharma, Shanghai, China). U6 snRNA was used as an endogenous control for miRNA detection. The expression of miR-200b was quantified by measuring cycle threshold (Ct) values and normalized using the 2-^ΔΔCt^ method relative to U6 snRNA.

### Patients and specimens for tissue microarray

A total of 134 female breast cancer patients who were diagnosed by histo-pathology in Sun Yat-Sen University Cancer Center from October 2001 to September 2006 were obtained. Specimens were formalin-fixed and embedded in paraffin by standard methodology after obtained during surgery and were stored in the Department of Specimen and Resource in Sun Yat-Sen University Cancer Center. IHC of ER, PR, and HER-2 status were performed in the Pathology Department of Sun Yat-Sen University Cancer Center. All the patients included in present study did not receive any chemotherapy and radiation therapy before, and their complete clinico-pathological data, including age, histological type, lymph nodes status, tumor size, stage, local relapse, distant metastatic relapse, ER status, PR status and HER-2 status, were available and reviewed. Histological type, reclassified according to the WHO classification and stage of tumor, was based on the TNM staging system (American Joint Committee on Cancer classification). Follow-up was updated by review of records and telephone calls. The date of death and the date of relapse were used to calculate estimate overall survival (OS) and disease-free survival (DFS).

This study was approved by the Ethics Committee of SunYat-Sen University Cancer Center Health Authority. The collection and use of tissues followed the procedures that are in accordance with the ethical standards as formulated in the Helsinki Declaration.

### Tissue microarray (TMA) construction

Representative part of the breast cancer specimens used for creating tissue microarray were selected by two experienced pathologists, using hematoxylin and eosin–stained sections which were formalin-fixed and embedded in paraffin as mentioned above. TMA block was constructed with MiniCore Control Station (ALPHELYS SARL, France) and designed by TMA Designer tissue array design software (ALPHELYS SARL, France). We used 1.0-mm core tissue biopsies and took tissues from paraffin-embedded tissue blocks to two new recipient blocks (one contained 51 samples, and the other 83 samples), and one core per case was arrayed. The recipient blocks were cut and placed on slides.

### LNA probes for miR-200b

miR-200b miRCURYTM LNA custom detection probe (Exiqon, Vedbaek, Denmark) was used for ISH. The 5′-3′ sequences (enhanced with LNA) were TCATCATTACCAGGCAGTATTA with a digoxigenin (DIG) label at both the 5′ and 3′ ends.

### In situ hybridization of miR-200b and scoring system

After deparaffinized, the slides were mounted onto flow through slide chambers and placed in a Tecan Freedom Evo automated hybridization instrument (Tecan, Männedorf, Switzerland) in which the following steps were performed: proteinase-K treatment 15 μg/ml at 37°C for 8 min, pre-hybridization in Exiqon hybridization buffer (Exiqon, Vedbæk, Denmark) at 62°C for 15 min, hybridization with 40 nM miR-200b probe, stringent washes with 5 × SSC, 1 × SSC and 0.2 × SSC buffers at 62°C over 33 min, DIG blocking reagent (Roche, Mannheim, Germany) in maleic acid buffer containing 2% sheep serum at 30°C for 15 min, Streptavidin-HRP -conjugated anti-digoxigenin (diluted 1:500 in blocking reagent, Roche) at 30°C for 30 min. After washing, the slides were incubated in DAB at 25°C for 3-5 min. The slides were then dismantled in water, dehydrated in alcohol solutions and mounted with eukitt mounting medium (VWR, Herlev, Denmark).

The intensities of miR-200b staining was scored by 0–4, according to the standards of 0–1 (no staining), 1–2 (weak staining), 2–3 (medium staining) and 3–4 (strong staining). The percentages of miR-200b cells of individual samples were analyzed. Those expression scores equaled to the intensities * the percentages, and the maximum was 4 and the minimum was 0. Individual samples were evaluated by at least two pathologists in a blinded manner, and those expression scores of greater than 2 were defined high expression, otherwise the expression scores were low.

### Immunohistochemical (IHC) staining and scoring system

The Labeled StreptAvidin Biotin Method was used for IHC in our study. After deparaffinizing and rehydrating, the slides were treated with 90% methanol/3% H_2_O_2_ solution for 15 min at room temperature to block endogenous peroxidase. Then, the slides were soaked in sodium citrate buffer (10 mM Sodium citrate, 0.05% Tween 20, pH 6.0) under 96°C for 5 min for antigen retrieval. After blocking by BSA, the following antibodies were used: mouse monoclonal antibody for RAB21 (sc-81917), rabbit polyclonal antibody for RAB23 (sc-130248) and RAB3B (sc-305), goat polyclonal antibody for RAB18 (polyclonal antibody for RAB) (Santa Cruz Biotechnology). We added antibodies to the slides for overnight storage at 4°C and then incubated the slides at room temperature with biotinylated secondary antibody for 20 min, and finally HRP-Streptavidin for 15 min. After DAB staining, the results were graded for intensity (0-negative, 1-weak, 2-moderate, and 3-strong) and percentage of positive cells (0, 1 (1–24%), 2 (25–49%), 3 (50–74%), and 4 (75–100%)) with discrepancies resolved by consensus. The grades were multiplied to determine a score. The scores of tumors were defined as the following rule: negative (score = 0–3) and positive (score > =4).

### Construction of luc-UTR vectors

The full-length RAB21, RAB23, RAB18, RAB3B, RAB37, RAB8B, RAB7A, RAP1B, RAP2C 3′-UTR was cloned into the EcoRIand HindIII sites of the pMIR-REPORT luciferase vector (Ambion, Austin, TX, U.S.) using PCR generated fragment. A Luc-mut vector of RAB21, RAB23, RAB18, and RAB3B in which the first seven nucleotides complementary to the miR-200b seed-region were mutated by site-directed mutagenesis (Stratagene) served as a mutant control.

### Luciferase assay

Luc-wt, Luc-mut, and Luc-ctrl were co-transfected within vitro-produced miR-200b into MDA-MB-231 cells. The pMIR-REPORT β-galactosidase control vector was transfected and served as a control. Luciferase activity was measured in cell lysates 48 h after transfection using a dual-light luminescent reporter gene assay kit (Applied Biosystems). Results were normalized against β-galactosidase activity.

### Western blot

Cell protein lysates, cytosol protein or nuclear protein was separated in 10% SDS-polyacrylamide gels, electrophoretically transferred to polyvinylidene difluoride membranes (Millipore), then detected with mouse monoclonal antibody for RAB21 (sc-81917), rabbit polyclonal antibody for RAB23 (sc-130248) and RAB3B (sc-305), goat polyclonal antibody for RAB18 (polyclonal antibody for RAB) (Santa Cruz Biotechnology), mouse monoclonal antibody for β-actin (Abcam) and commercial ECL kit (Pierce). The intensity of protein fragments was quantified using Chemical DocTM XRS + (Bio-Rad).

### RNA silencing for RAB21, RAB23, RAB18, and RAB3B

The sense sequences of siRNA oligonucleotides targeting the RAB18, RAB21, RAB23, and RAB3B transcripts, respectively, were as follows: si- RAB18: 5′-UUCUGGUUGUAACUUCACGGCT-3′; si- RAB21: 5′-UUAAUAGGUUGGAUGGCGGTT-3′; si- RAB23: 5′-CUUCACTACUGCUUCGAGTT-3′; and si- RAB3B: 5′-AUAACUUGGAGGGACUGCCTT-3′ (Invitrogen). Scrambled siRNA was used as a negative control. Cells were plated in culture dishes or in 24-well plates for 24 h, and transfected with siRNA using Lipofectamine 2000. After 48 h, the cells were harvested for use in other assays.

### MTT assay

Cell viability was examined by the 3-(4, 5-dimethylthiazol-2-yl)-2, 5-diphenyltetrazolium bromide (MTT) assay. Cells transfected with either scramble or miR-200b mimics were seeded at a density of 5,000 cells per well in 96-well plates and incubated at 37°C for 24 h. Cells were then incubated an additional 72 h, and the MTT assay was performed according to the manufacturer’s instructions (Molecular Probes, Eugene, OR). Absorbance values were determined at 570 nm on a Spectra Max 250 spectrophotometer (Molecular Devices, Sunnyvale, CA).

### Cell invasion and migration assays

The cell invasion assay was conducted as described previously [18]. Briefly, cells were seeded onto the basement membrane matrix present in the insert of a 24-well culture plate (EC matrix, Chemicon, Temecula, CA). Fetal bovine serum was added to the lower chamber as a chemoattractant. After an additional 48 hours, the non-invading cells and EC matrix were gently removed with a cotton swab. Invasive cells located on the lower side of the chamber were stained with crystal violet, counted and imaged.

### Statistical analysis

Data are presented as mean ± SD from at least three separate experiments. Multiple group comparisons were performed using ANOVA with a post hoc test for subsequent individual group comparisons. The distinct expression of miR-200b between tumor tissues and para-carcinoma tissues was examined by independent samples *T*-test. The relationships between miR-200b expression and clinico-pathological parameters were examined by chi-square test. Overall survival (OS) or disease-free survival (DFS) curves were calculated by the Kaplan-Meier method and the log-rank test was used to determine the difference in OS or DFS rates between two groups. Results were considered statistically significant when *P* ≤ 0.05 was obtained. All the statistical analyses were performed using SPSS13.0 for Windows (SPSS Inc., Chicago, IL, USA).

## Results

### The expression of miR-200b in breast cancer tissues and cell lines

**Figure 1:**
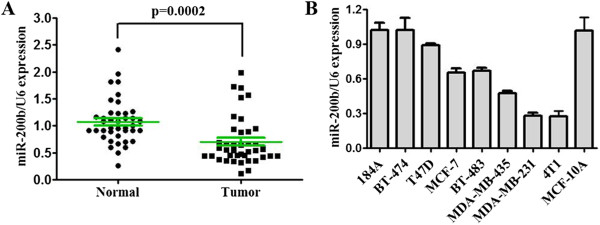
**miR-200b was down-regulated in breast cancer tissues and cell lines. (A)** The expression of miR-200b was detected by qRT-PCR in 40 pairs of normal mammary tissues and breast cancer samples. **(B)** The expression of miR-200b was detected by qRT-PCR in breast cancer cell lines.

To evaluate the expression of miR-200b in breast cancer tissues, qRT-PCR was used to detect the expression level in 40 pairs of tumor tissues and para-carcinoma (normal) tissues from breast cancer patients. The results showed that expression of miR-200b in breast cancer tissues was significantly lower than in normal tissues (Figure 1A). In comparison with the normal tissues, miR-200b was down-regulated in 77.5% (31/40) of the tumor samples. We further detected expression of miR-200b in cell lines (Figure 1B), including human mammary epithelial (HME) cell lines (184A, MCF-10A), human breast cancer cell lines (MDA-MB-231, MDA-MB-435, MCF-7, T47D, BT-474, BT-483), and mouse breast cancer cell line 4 T1. Compared with 184A and MCF-10A, miR-200b was down-regulated in MCF-7, MDA-MB-435, T47D, BT-483, MDA-MB-231, and 4 T1, especially in the latter two cell lines. These results revealed that reduced miR-200b expression was a frequent event in human breast cancer tissues and could be involved in breast cancer carcinogenesis.

### The relationships between expression of miR-200b and clinical parameters in breast cancer patients

**Figure 2:**
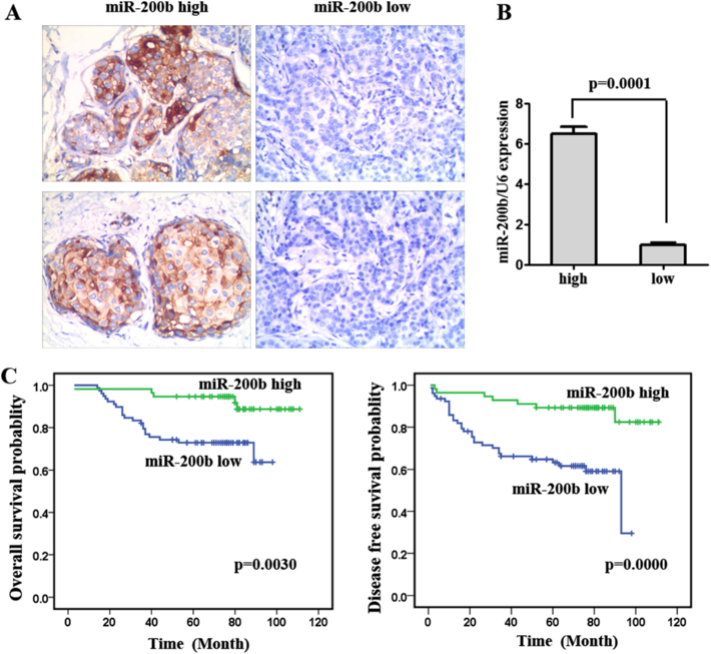
**miR-200b as a prognostic factor in breast cancer patients. (A)** The ISH staining of miR-200b in breast cancer tissue microarray: ×200. Two miR-200b high samples and two miR-200b low samples were shown as representatives. **(B)** qRT-PCR results for miR-200b detection in three miR-200b high ISH staining samples and three miR-200b low samples. **(C)** Survival curves of OS and DFS according to miR-200b expression. Low miR-200b expression correlated with worse outcome.

We further evaluated the prognostic role of expression of miR-200b in breast cancer patients by in situ hybridization (ISH) staining on tissue microarray. MiR-200b ISH staining in breast cancer tissues was located in the cytoplasm. Two miR-200b high samples and two miR-200b low samples were shown as representatives (Figure 2A). To confirm the miR-200b ISH staining results, we extracted total RNAs form three miR-200b high samples and three miR-200b low samples for qRT-PCR (Figure 2B). The PCR results showed that the ISH staining could reflect the relative level of miR-200b expression in the samples. The clinico-pathologic characteristics and miR-200b expression of the breast cancer patients involved in our study are shown in Table 1. In all 134 breast cancer patients, high expression of miR-200b was seen in 41.8% of total patients. High miR-200b expression correlated with early TNM stage (p = 0.040) and fewer metastasis (p = 0.003). Furthermore, markedly reduced overall survival (p = 0.0030) and disease-free survival (p = 0.0000) were observed in the breast cancer patients who had low miR-200b expression compared with the patients who exhibited high expression levels (Figure 2C). In our study, the estimate overall survival and disease-free survival for miR-200b high patients were 109.3 months and 101.2 months, while the two for miR-200b low patients were 78.3 months and 66.2 months respectively. These results indicated that miR-200b could be a prognostic factor in breast cancer patients.

**Table 1:** Clinico-pathological variables and the expression of miR-200b in total breast cancer patients

**Characteristics**	**Total (n = 134)**	**miR-200b low (n = 78)**		**miR-200b high (n = 56)**		**P value**
		**No.**	**%**	**No.**	**%**	
Age (years)						0.330
<50	82	46	56.1	36	43.9	
>50	52	32	61.5	20	38.5	
Menopause						0.524
Yes	63	37	58.7	26	41.3	
No	71	41	57.7	30	42.3	
LN infiltrated						0.148
Yes	80	50	62.5	30	37.5	
No	54	28	51.9	26	48.1	
Tumor size (cm)						0.080
= < 2	38	18	47.3	20	52.7	
>2	96	60	62.5	36	37.5	
TNM stage						0.040*
I-II	78	40	51.3	38	48.7	
III- IV	56	38	67.9	18	32.1	
Local relapse						0.083
Yes	8	7	87.5	1	12.5	
No	126	71	56.3	55	43.7	
Distant metastasis						0.003*
Yes	34	27	79.4	7	20.6	
No	100	51	61.0	49	49.0	
ER status						0.155
Positive	51	33	64.7	18	35.3	
Negative	83	45	54.2	38	45.8	
PR status						0.073
Positive	54	36	66.7	18	33.3	
Negative	80	42	52.5	38	47.5	
HER-2 status						0.213
Positive	22	15	68.2	7	31.8	
Negative	112	63	56.3	49	43.8	
TNBC status						0.472
TNBC	51	29	56.9	22	43.1	
Non-TNBC	83	49	59.0	34	41.0	

*means statistically significant (p < 0.05).

% means percentage within the row.

TNBC, triple-negative breast cancer, means ER(-), PR(-), HER-2(-). High expression of miR-200b was seen in 41.8% of total patients and correlated with early TNM stage (p = 0.040) and fewer metastasis (p = 0.003).

### miR-200b targeted multiple members of RAB family

**Figure 3:**
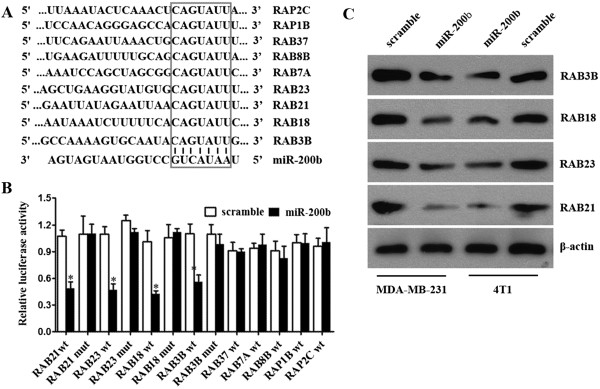
**RAB21, RAB23, RAB18 and RAB3B is a direct target of miR-200b. (A)** miR-200b binds to the 3′UTRs of RAB21, RAB23, RAB18, RAB3B, RAB37, RAB8B, RAB7A, RAP1B and RAP2C. Predicted binding between miR-200b and the seeds match in the nine predicted genes’ 3′UTRs. **(B)** Luciferase reporter assays 48 h after transfection with indicated pMIR-Report plasmids and a renilla transfection control plasmid, co-transfected with miR-200b mimics, or relevant scramble controls. Luciferase activities were significantly inhibited on co-transfection of miR-200b mimics and pMIR-Report plasmids of RAB21, RAB23, RAB18 and RAB3B. Mutation in the corresponding binding sites abolished this inhibition. Data shown were the mean ± s.d. of six replicates and were representative of three independent experiments. *P < 0.05 **(C)** miR-200b regulated the expression of RAB21, RAB23, RAB18 and RAB3B. Western blot analyzed their expression 48 h after transfection with miR-200b mimics or scramble control in MDA-MB-231 and 4 T1 cells.

To determine the function of miR-200b in breast cancer, we used online softwares TargetScan to search for potential miR-200b target genes. We found that members of RAB family are among these candidate target genes. A miR-200b-binding site was found in the 3′-UTR of RAB21, RAB23, RAB18, RAB3B, RAB37, RAB8B, RAB7A, RAP1B, RAP2C mRNA with perfect base pairing (Figure 3A). To verify whether these genes were direct targets of miR-200b, we subcloned the full-length 3′-UTR of RAB21, RAB23, RAB18, RAB3B, RAB37, RAB8B, RAB7A, RAP1B, RAP2C into the luciferase reporter vector. However, addition of in vitro-produced miR-200b only suppressed the luciferase activity of the 3′-UTR of RAB21, RAB23, RAB18 and RAB3B upon co-transfection of the luciferase vector (wild-type, mutant, or negative control) with the in vitro-produced microRNAs (miR-200b mimics or scramble control) into MDA-MB-231 cells (Figure 3B). This inhibition was abolished when the seed sequences of the miR-200b target sequences were mutated in the Luc-mut vector (Figure 3B). To directly assess the effects of miR-200b on the expression of RAB21, RAB23, RAB18 and RAB3B, we transfected miR-200b mimics into MDA-MB-231 and 4 T1 cells and found that overexpression of miR-200b reduced mRNA and protein level of RAB21, RAB23, RAB18 and RAB3B (Figure 3C).

### The association between expression of miR-200b and RAB21, RAB23, RAB18 and RAB3B in breast cancer tissues

**Figure 4:**
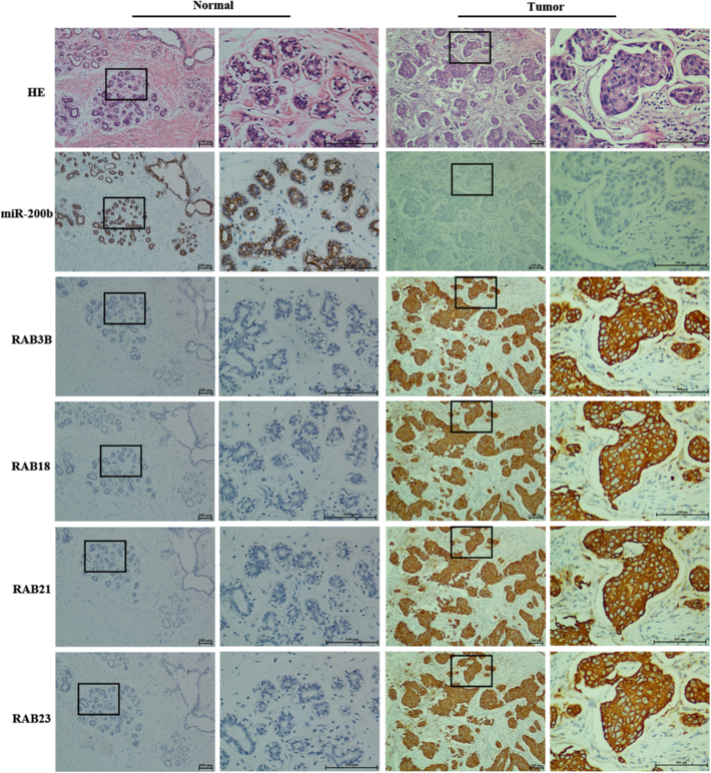
**IHC staining of RAB21, RAB23, RAB18 and RAB3B in human breast cancer tissues.** 10 pairs of tumor tissues and normal tissues from breast cancer patients, who had developed distant metastasis after operation, were detected by ISH or IHC. Compared with normal tissue, miR-200b expression was down-regulated in tumor tissue, while the contrary situations were found for RAB21, RAB23, RAB18 and RAB3B.

To evaluate the associations between expression of miR-200b and RAB21, RAB23, RAB18 and RAB3B in breast cancer tissues, we further detected expression of miR-200b by ISH staining and expression of RAB21, RAB23, RAB18 and RAB3B protein by immunohistochemistry (IHC) staining in 10 pairs of tumor tissues and para-carcinoma tissues from breast cancer patients, who had developed distant metastasis after operation. As shown in Figure 4, compared with normal breast tissue, miR-200b expression was down-regulated in tumor tissue, while the contrary situations were found for RAB21, RAB23, RAB18 and RAB3B IHC staining. In summary, miR-200b low ISH staining was seen in 90% (9/10) tumor samples, while positive rates for RAB21, RAB23, RAB18 and RAB3B IHC staining were 80%, 80%, 90%, 100% respectively. These results further confirmed the regulation of RAB21, RAB23, RAB18 and RAB3B proteins by miR-200b in breast cancer tissues.

### Over-expression of miR-200b or knock-down of RAB21, RAB23, RAB18 and RAB3B inhibited breast cancer cell proliferation and invasion

**Figure 5:**
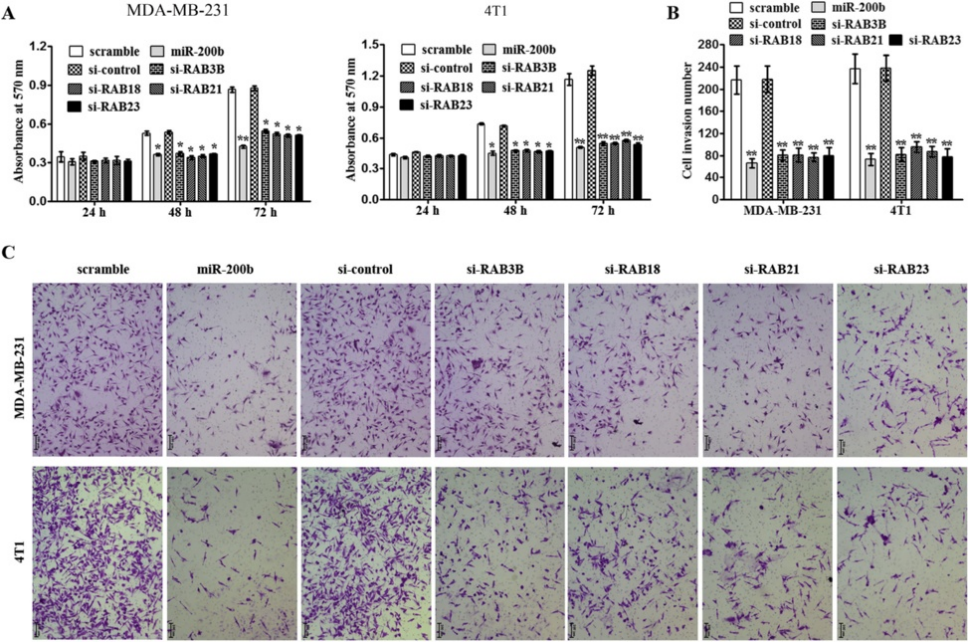
**Over-expression of miR-200b or knock-down of RAB21, RAB23, RAB18 and RAB3B inhibits breast cancer cell proliferation and invasion. (A)** Transfection of miR-200b mimics or knock-down of RAB21, RAB23, RAB18 and RAB3B in MDA-MB-231 and 4 T1 cells markedly attenuated cell proliferation: MTT assay at 24 h, 48 h and 72 h after transfection. **(B, C)** Transfection of miR-200b mimics or knock-down of RAB21, RAB23, RAB18 and RAB3B in MDA-MB-231 and 4 T1 cells inhibited cell invasion: Data shown in Figure B were the mean ± s.d. of three replicates and were representative of three independent experiments. *P < 0.05. The representing images were shown in Figure **C**.

To assess the biological effects of over-expressing miR-200b in breast cancer cells, ectopic expression of miR-200b mimics were transfected into MDA-MB-231 and 4 T1 cells. Transfection of miR-200b mimics in MDA-MB-231 and 4 T1 cells markedly attenuated cell proliferation, compared with scramble (Figure 5A). Moreover, ectopic expression of miR-200b mimics in MDA-MB-231 and 4 T1 cells markedly attenuated cell invasion compared with control cells (Figure 5B, C). To identify the biological effects of RAB21, RAB23, RAB18 and RAB3B, specific siRNAs for each were also synthesized. Transfection of siRNAs for RAB21, RAB23, RAB18 and RAB3B also inhibits breast cancer cell proliferation and invasion (Figure 5A, B, and C). These results indicated that the biological effects of miR-200b in breast cancer cells may attribute to regulation of RAB21, RAB23, RAB18 and RAB3B, and thus miR-200b could be a therapeutic target in breast cancer.

## Discussion

The miR-200 family has been reported to be a fundamental regulator of EMT, thus highlighting their roles in cancer progression. As a founding member in miR-200 family, miR-200b attracts much focus both in carcinogenesis and cancer therapy in recent years [17]. Down-regulation of miR-200b has been observed in renal cell carcinoma [19]. miR-200b as tumor suppressor regulating EMT has bee reported in several malignancies, such as prostate cancer [20], colon cancer [21], non-small cell lung cancer [22], and so on. The dysregulation of miR-200b in cancer could be transcriptional inhibition or epigenetic modifications, such like DNA methylation and histone modifications [23,24]. Moreover, miR-200b is thought to be related to cell differentiation by targeting GATA-4 [25]. Loss of miR-200b contributes to the breast cancer stem cell status maintaining [26]. Besides, miR-200b has also been associated with cancer chemo-sensitivity by modulating PTEN, PTPN12 and thus their downstream oncogenes like src and ras [27,28].

In this study, we investigated the expression of miR-200b and its prognostic role in breast cancer. We showed that expression of miR-200b was significantly lower in breast cancer tissues than normal tissues. Similar results were found in breast cancer cell lines. In addition, we analyzed the prognostic role of the expression of miR-200b in breast cancer patients. We found that low expression of miR-200b correlated with advanced clinical stage and more distant metastasis in breast cancer. Since miR-200b was reported as a regulator of EMT, which was thought to be the initiation of metastasis, the results were reasonable. Further we showed that the patients with low miR-200b expression correlated with worse outcome, indicating miR-200b as a tumor suppressor in breast cancer.

We predicted the targets of miR-200b in breast cancers. Besides previously reported ZEB1/ZEB2, we found multiple members of RAB family were also potential targets of miR-200b. We then performed the luciferase report assay to identify RAB21, RAB23, RAB18 and RAB3B as novel direct targets of miR-200b. The regulations of RAB21, RAB23, RAB18 and RAB3B by miR-200b were further confirmed in breast cancer cell lines.

RAB family proteins belong to the large Ras superfamily of small GTPases [29]. There are more than 60 members in RAB family in humans, which are specifically localized to subcellular membrane compartments, regulating intracellular membrane transport. By switching between inactive cytosolic GDP-bound forms and active membrane-associated GTP-bound forms, RAB proteins could control endocytosis, protein secretion, recycling and degradation, thus acting as key regulators of intracellular trafficking [30]. Recent studies have provided emerging evidences for involvement of RAB proteins in tumour progression. More and more members of RAB family have been reported to be dysregulated in cancers, either as oncogenes or tumor suppressors [31]. Several RAB proteins have been linked with tumor migration, invasion and drug resistance. Among them, up-regulation of Rab3B is reported in prostate cancer, promoting cancer cell survival [32]. Rab21 is associated with the control of integrin trafficking, thus modulating adhesion and motility in breast cancer cells [33]. Another RAB family member, RAB23 has been identified as an antagonist of Sonic Hedgehog signaling pathway which is deregulated in many cancers [34], and found to over-expressed in gastric and liver cancer [35].

In our study, we identified RAB21, RAB23, RAB18 and RAB3B as novel direct targets of miR-200b in breast cancers. Over-expression of miR-200b or knock-down of the four RAB proteins significantly inhibited breast cancer cell proliferation and invasion. However, whether the tumor suppressing effects of miR-200b in breast cancer depends dominatingly or partially on its regulation of RAB proteins needs more investigation.

## Conclusion

In summary, our study demonstrated that miR-200b could be a tumor suppressor and a potential biomarker in breast cancer patients. Members of RAB family, RAB21, RAB23, RAB18 and RAB3B were novel targets regulated by miR-200b in breast cancer, which could be of promising therapeutic significance.

## Competing interests

No potential conflicts of interest were disclosed.

## Authors’ contributions

FY and HT designed the experiments, interpreted the data, and wrote the manuscript. FY, HT and QL carried out experiments. FY, HT, QL, XX, MW, XL, BC collected the human samples and clinical data. All authors read and approved the final manuscript.
